# Comparative Transcriptomics Analysis Reveals Unique Immune Response to Grass Carp Reovirus Infection in Barbel Chub (*Squaliobarbus curriculus*)

**DOI:** 10.3390/biology13040214

**Published:** 2024-03-25

**Authors:** Yuhong Huang, Xiaodong Wang, Zhao Lv, Xudong Hu, Baohong Xu, Hong Yang, Tiaoyi Xiao, Qiaolin Liu

**Affiliations:** 1 Fisheries College, Hunan Agricultural University, Changsha 410128, China; yuhonghuang0619@163.com (Y.H.); wangxd1225@126.com (X.W.); lvzhao_0320@hunau.edu.cn (Z.L.); huxudong@hunau.edu.cn (X.H.); xbht568@126.com (B.X.); hongyang1920@163.com (H.Y.); 2 Yuelushan Lab, Changsha 410128, China

**Keywords:** GCRV, comparative transcriptome, antiviral immunity, PRRs, cytokine, complement and coagulation cascade, apoptosis, autophagy

## Abstract

**Simple Summary:**

GCRV has severely threatened the grass carp cultivation industry. However, barbel chub, which share a close relationship with grass carp, have shown strong resistance to GCRV infection. To explore the reasons for GCRV resistance in barbel chub, we constructed gene expression profiles of its main immune tissues (i.e., liver, spleen, head kidney, and trunk kidney) after GCRV infection. We compared the transcriptome of grass carp and barbel chub and found that barbel chub respond to GCRV infection via PRRs, cytokine-related pathways, complement and coagulation cascades, apoptosis, and autophagy. Our study provides evidence that these pathways might be potential reasons for the GCRV resistance of barbel chub.

**Abstract:**

Grass carp (*Ctenopharyngodon idella*) and barbel chub (*Squaliobarbus curriculus*)—both Leuciscinae subfamily species—demonstrate differences in grass carp reovirus (GCRV) infection resistance. We infected barbel chubs with type II GCRV and subjected their liver, spleen, head kidney, and trunk kidney samples to investigate anti-GCRV immune mechanisms via RNA sequencing and quantitative real-time polymerase chain reaction (qRT-PCR). We identified 139, 970, 867, and 2374 differentially expressed genes (DEGs) in the liver, spleen, head kidney, and trunk kidney, respectively. Across all four tissues, gene ontology analysis revealed significant immune response-related DEG enrichment, and the Kyoto Encyclopedia of Genes and Genomes analysis revealed pattern recognition receptor (PRR) and cytokine-related pathway enrichment. We noted autophagy pathway enrichment in the spleen, head kidney, and trunk kidney; apoptosis pathway enrichment in the spleen and trunk kidney; and complement- and coagulation-cascade pathway enrichment in only the spleen. Comparative transcriptome analysis between GCRV-infected barbel chubs and uninfected barbel chubs comprehensively revealed that PRR, cytokine-related, complement- and coagulation-cascade, apoptosis, and autophagy pathways are potential key factors influencing barbel chub resistance to GCRV infection. qRT-PCR validation of 11 immune-related DEGs confirmed our RNA-seq data’s accuracy. These findings provide a theoretical foundation and empirical evidence for the understanding of GCRV infection resistance in barbel chub and hybrid grass carp–barbel chub breeding.

## 1. Introduction

As lower vertebrates, fish have evolved a sophisticated immune system, both innate and adaptive. In contrast to that in mammals, the adaptive immune system in fish is imperfect. Consequently, innate immunity plays a major role in defense against pathogenic invasions in fish [1]. In the case of pathogen invasion, pattern recognition receptors (PRRs), the sentinels of the innate immune system, recognize pathogen-associated molecular patterns (PAMPs) and activate the host innate immune response [2]. This process involves the activation of immune molecules, such as cytokines and complements, to combat pathogen invasion. Cytokines—small protein molecules secreted by immune cells—are vital communicators between cells, performing immune regulation and effector functions [3]. The complement and coagulation cascades are pivotal in immune response in both mammals and fish because they can induce inflammation, eliminate infected cells, and activate adaptive immunity [4,5]. In addition to combating pathogen invasion via immune molecule activation, cells can initiate immune mechanisms, such as autophagy and apoptosis, to resist pathogen invasion. Autophagy, a crucial biological process, is key in maintaining the balance of inflammatory and immune responses via the efficient capture and transport of cargo (including PAMPs and damage-associated molecular pattern molecules) in the cytoplasm to lysosomes for degradation [6]. Moreover, apoptosis plays a major role in the pathogen infection process. Pathogen infection triggers programmed cell death, after which apoptotic cells are rapidly and effectively efferocytosed by both professional and nonprofessional phagocytic cells [7]. As a whole, innate immunity is a crucial component of the fish immune defense system, involving various immune responses collectively participating in resistance against pathogens.

Grass carp is a freshwater aquaculture species with the highest yield in China and with considerable economic value. However, the development of the grass carp aquaculture industry is severely limited by grass carp hemorrhage disease. Grass carp hemorrhage disease is characterized by high infectivity and mortality, which causes substantial economic loss to the grass carp aquaculture industry annually [8]. Grass carp hemorrhage disease is caused by grass carp reovirus (GCRV), which can also lead to infections in species such as barbel chub, stone moroko (*Pseudorasbora parva*), and rare minnow (*Gobiocypris rarus*) [9]. In both stone moroko and rare minnow, GCRV infection is associated with relatively high mortality; in contrast, it is associated with relatively low mortality in barbel chub [9,10]. Taxonomically, both barbel chub and grass carp belong to the Leuciscinae subfamily; as such, they share a relatively close relationship. Consequently, our group conducted preliminary experiments on grass carp–barbel chub hybridization. The results demonstrated that the hybrid progeny exhibited resistance to GCRV infection, possibly because the hybrid offspring inherited GCRV resistance genes from the barbel chub [10]. In summary, barbel chub may be a donor of resistance gene resources and resistance mechanisms in the hybrid offspring. However, the response characteristics and molecular mechanisms underlying GCRV infection in barbel chub, as well as the differences in disease resistance between barbel chub and grass carp, remain unclear.

Several studies have reported the molecular mechanisms underlying immunity to GCRV infection in barbel chub. For instance, after GCRV infection, grass carp and barbel chub exhibit differences in melanoma differentiation-associated gene 5 (*MDA5*) expression patterns. After barbel chubs are infected with GCRV, *MDA5* expression is significantly upregulated in the liver and spleen; it eventually returns to nearly normal levels. The duration of sustained MDA5 upregulation after GCRV infection is longer in grass carp than in barbel chubs [11,12]. Furthermore, the expression of barbel chub toll-like receptor 7 (*TLR7*) and toll-like receptor 8 (*TLR8*) remains upregulated after GCRV infection [13]. In grass carp, *TLR7* expression is upregulated over 6 h after initiation of GCRV infection; however, it is downregulated thereafter [14]. These findings suggest that the differences in PRR expression patterns between grass carp and barbel chub might be a reason for the variance in their disease resistance levels. Moreover, complement gene expression is significantly higher in barbel chub than in grass carp [15]. Furthermore, transcriptome analysis of grass carp spleen shows that the expression of various complement components was downregulation after GCRV infection [16]. Therefore, the differences in complement protein expression and function between grass carp and barbel chub may also reveal the differences in their resistance to GCRV infection.

Although the aforementioned studies have elucidated some differences between the immune responses to GCRV in grass carp and barbel chub, their results are inconsistent and nonsystematic. In this study, to systematically delineate the mechanisms underlying the differences in resistance to GCRV between grass carp and barbel chub, we employed comprehensive transcriptomic analysis to examine the gene transcription profiles in the main immune tissues (i.e., liver, spleen, head kidneys and trunk kidneys) of barbel chub after GCRV infection. The current findings revealed a unique immune response mechanism to GCRV infection in barbel chub, indicating that multiple immune processes play a collaborative role in the immune response. These immune processes include complement and coagulation cascades, PRRs, cytokine-related pathways, apoptosis, and autophagy. These results demonstrate a reaction pattern that actively responds to the host immune system and maintains a balance between inflammation and immunity. Collectively, this study improves the current understanding of differences in GCRV resistance between grass carp and barbel chub, providing a theoretical basis for future selection breeding of grass carp with disease resistance and experimentation of hybrid breeding between grass carp and barbel chub.

## 2. Materials and Methods

### 2.1. Animals and Virus

This animal study was reviewed and approved by the Institutional Animal Care and Use Committee (IACUC) of Hunan Agricultural University. By using a protocol outlined by Shi et al. [17], a suspension of GCRV (GCRV104) from diseased grass carp with typical symptoms was prepared. Healthy barbel chub (weight = 50 ± 5 g) were sourced from the fishery base of Hunan Agricultural University, Hunan Province, China. The fish were acclimated in a recirculating water system containing freshwater at 28 °C for 2 weeks. The commercial fish feed (Haid group, Guangzhou, China) was used as basic feed. They were fed at 2% of their body weight twice a day (at 8:00 a.m. and 5:00 p.m.). 180 barbel chub were randomly divided into control and experimental groups (n = 30 fish/tank, with 3 tanks per group). Three tanks of experiment group, one for transcriptome analysis and two for infection survival. The treatment of the control group was the same as that of the experimental group. GCRV infection was initiated via feeding. The specific infection methods referred to the protocol outlined by Wang et al. [18].

### 2.2. Sample Collection, RNA Extraction, and Library Construction

Five days after GCRV administration, we selected three fish from the experimental group and three from the control group. These fish were euthanized using an overdose of tricaine methane sulfate (MS222, 200 mg/L). Subsequently, their liver, spleen, and head and trunk kidneys were harvested, immediately frozen in liquid nitrogen, and stored at −80°C until further analysis.

Tissue total RNAs were extracted using TRIzol reagent (Invitrogen, Waltham, MA, USA), according to the manufacturer’s instructions. To verify RNA integrity, we performed 1% agarose gel electrophoresis and checked for RNA degradation and contamination. RNA concentration was quantified using a NanoDrop 2000 spectrophotometer (Thermo Fisher Scientific, Waltham, MA, USA), and RNA integrity was assessed on an Agilent 2100 Bioanalyzer with the RNA Nano 6000 Assay Kit (Agilent Technologies, Santa Clara, CA, USA). mRNA was purified from total RNA by using oligo (dT) magnetic beads. Divalent cations were used to fragment DNA at elevated temperatures with NEBNext First Strand Synthesis Reaction Buffer (NEB). RNase H was used to reverse-transcribe RNA to first-strand cDNA by using random hexamer primers. RNase H and DNA polymerase I were then used to synthesize second-strand cDNA. After the adenylation of the 3′ ends of DNA fragments using the NEBNext Adaptor, hybridization preparation was completed. To select cDNA fragments of the preferred length (150–200 bp), the library fragments were purified using AMPure XP. The final step involved sequencing the library on the Illumina HiSeq 2500 system, and 250-bp paired-end reads were generated.

### 2.3. Transcriptome Assembly and Statistical Analysis

Cleaning the raw data involved removing adapters, poly-N sequences, and low-quality bases. The Q20, Q30, GC-content, and sequence-duplication levels of the cleaned data were calculated. The data used for downstream analyses was clean and high quality. The clean reads were assembled de novo in Trinity [19], with all other parameters set to default and min_kmer_cov set to 2 (by default). The gene functional data from Unigene was annotated and aligned against NCBI non-redundant protein sequences (NR), Protein family (Pfam), Clusters of Orthologous Groups of proteins (KOG/COG/eggNOG), Swiss-Prot (a manually annotated, reviewed protein sequence database), Kyoto Encyclopedia of Genes and Genomes (KEGG), and Gene Ontology (GO) database data. Read counts for each gene were obtained from the mapping results of each clean read using RSEM, as described previously [20].

### 2.4. Differentially Expressed Gene Analysis

Differentially expressed gene (DEG) analysis on four tissues in the two groups was performed using edgeR—which provides statistical procedures to determine differential expression in digital gene expression data by using a model based on the negative binomial distribution. *p* values were adjusted to control for the false discovery rate using the Benjamini–Hochberg method; moreover, adjusted *p* < 0.05 and log_2_[fold change (FC)] > 1.5 were used to identify DEGs. GO enrichment analysis of DEGs was performed using the Kolmogorov–Smirnov test with the R package topGO (Corrected *p*-Value < 0.01). The KEGG is a database resource for understanding the high-level functions and utilities of biological systems. We employed KOBAS to assess DEG enrichment within KEGG pathways statistically. A KEGG pathway with adjusted *p* < 0.05 was considered to indicate significant enrichment.

### 2.5. Quantitative Reverse Transcription Polymerase Chain Reaction for Gene Expression Profile Verification

Healthy barbel chub (weight = 3 ± 0.3 g) were acclimated in a recirculating water system in freshwater at 28 °C for 2 weeks. The fish were fed twice daily, at 8:00 a.m. and 5:00 p.m., at a rate of 2% of their body weight. For the infection study, healthy fish were immersed in a 40-fold diluted GCRV (GCRV104) suspension for 20 min. After 120 h of immersion, samples from the four tissues (the liver, spleen, head kidney, and trunk kidney) were collected. Total RNA was extracted using RNA isolate with Total RNA Extraction Reagent (Vazyme, Nanjing, China), according to the manufacturer’s instructions. To eliminate genomic DNA, 1 μg of the isolated RNA was treated with DNase I (Thermo Scientific, USA). The RevertAid First Strand cDNA Synthesis Kit (Thermo Scientific, USA) was used next to synthesize first-strand cDNA from 1 μg of RNA with oligo(dT)18 primers. This cDNA was used as the template for subsequent quantitative reverse transcription polymerase chain reaction (qRT-PCR). qRT-PCR was performed on a CFX96 Touch real-time PCR system by using SYBR Green qPCR Master Mix. The reaction mixture included 5 μL of the Master Mix, 3.2 μL of ddH_2_O, 0.4 μL each of forward and reverse primers, and 1 μL of cDNA. Gene-specific primers were designed using the Blast primer software program, based on gene sequences identified from the transcriptome library. β-Actin was used as the endogenous reference gene. The sequences of primers are listed in Table S1. qRT-PCR cycling conditions included denaturation at 95 °C for 2 min, followed by 40 cycles of amplification (i.e., 95 °C for 5 s and 60 °C for 15 s); next, melting curve analysis was performed at 95 °C for 5 s, 65 °C for 5 s, and an increase in temperature from 65 °C to 95 °C in 0.5 °C increments. Gene expression levels were quantified using Bio-Rad (Hercules, CA, USA) CFX Manager. All experiments were repeated at least three times, with the data being represented as the means ± standard errors of the means. Statistical significance was set at *p* < 0.05 (*). Heatmaps were created using GraphPad Prism (version 8).

## 3. Results

### 3.1. Transcriptome Assembly and Quality Assessments

**Figure 1:**
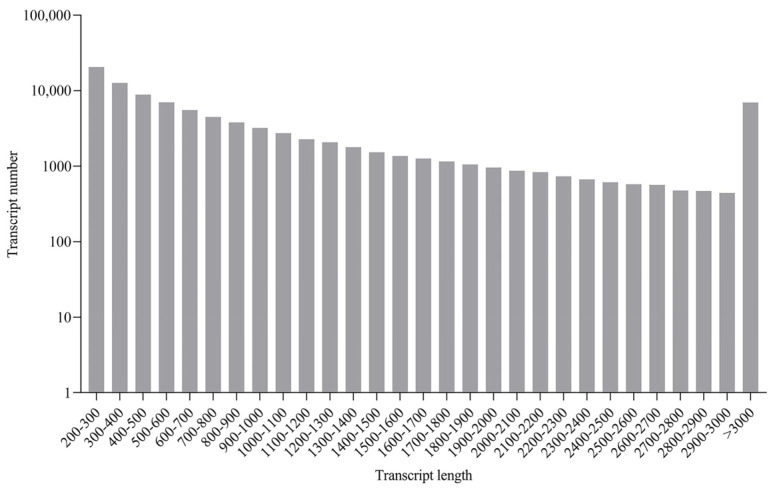
Sequence length distribution of contigs. The *x*- and *y*-axes indicate the transcript length (nt) and assembled transcript number, respectively.

RNA sequencing was conducted for the liver, spleen, head kidney, and trunk kidney from GCRV-infected barbel chubs and uninfected barbel chub. For sequence data analysis, raw reads were filtered to remove adaptor sequences and low-quality reads. In total, 175.38 Gb of clean data was obtained, with the clean data of each sample exceeding 6.14 Gb. In the absence of a reliable reference genome for barbel chub, these clean data were used to construct a unigene library. The distribution of sequence lengths from the contigs is illustrated in Figure 1. The transcriptome had an average length of 1506 bp and contained 95,657 unigenes, with an average unigene length of 1053 bp. Table S2 lists the clean read, clean bases, GC contents, Q30 values, and mapped rates for each library. All indicators demonstrated that the high quality of the sequencing data could be used for further analysis (Table S2). The data from this study have been successfully submitted to the NCBI Sequence Read Archive database (accession number: PRJNA1061050).

### 3.2. DEG Identification

**Figure 2:**
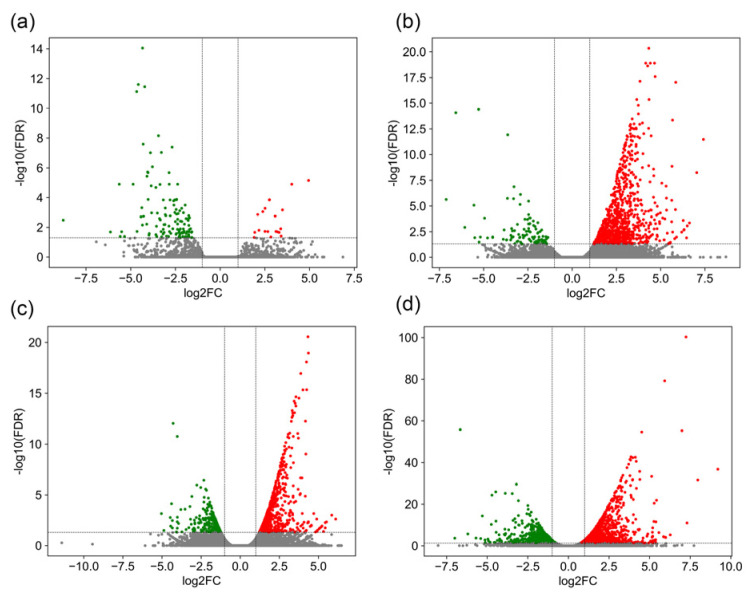
Volcano plots of DEGs in the liver (**a**), spleen (**b**), head kidney (**c**), and trunk kidney (**d**) after GCRV infection. The *x*-axis represents log_2_(FC), where a higher absolute value indicates greater expression differences, whereas the *y*-axis represents −log_10_(adjusted *p*), where a higher value indicates more reliable differential expression. Red represents upregulated genes and green represents downregulated genes.

A comprehensive differential expression analysis of GCRV-infected barbel chub revealed considerable changes in gene expression across various tissues. The results demonstrated 139 DEGs (20 upregulated and 119 downregulated) in the liver, 970 DEGs (869 upregulated and 101 downregulated) in the spleen, 867 DEGs (653 upregulated and 214 downregulated) in the head kidney, and 2374 DEGs (1565 upregulated and 809 downregulated) in the trunk kidney (Table S3). Figure 2 presents a volcano plot providing a visual representation of these DEGs, highlighting the predominance of downregulation in the liver and that of upregulation in the spleen, head kidney, and trunk kidney.

### 3.3. GO Analysis of DEGs

**Figure 3:**
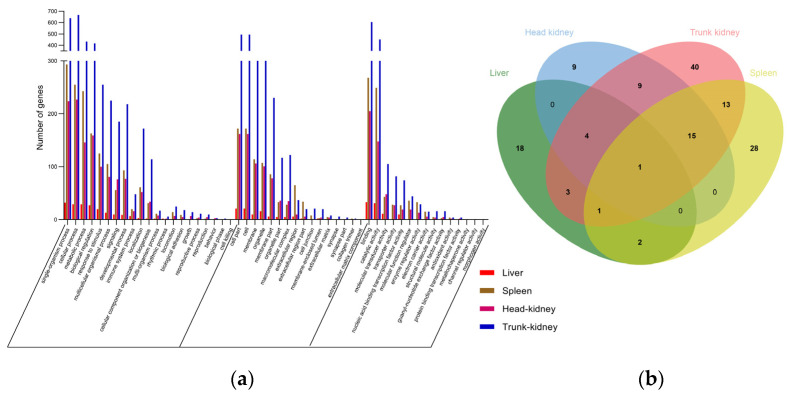
(**a**) GO classification of DEGs in the liver, spleen, head kidney, and trunk kidney after GCRV infection. The *x*-axis represents GO ontology; the functions are classified based on biological processes, cellular components, and molecular functions. The *y*-axis represents numbers of genes. (**b**) Venn diagram of immune-related GO terms in the liver (green), spleen (blue), head kidney (red), and trunk kidney (yellow) after GCRV infection. The values represent the numbers of GO terms in the corresponding tissues.

We used the GO database to categorize the DEG functions into three main clusters: biological processes, cellular components, and molecular functions. All DEGs were distributed across 49 GO categories (Figure 3a), with liver, spleen, head kidney, and trunk kidney DEGs being distributed across 32, 44, 50, and 48 GO categories, respectively. The GO categories are listed in Table S4.

#### 3.3.1. Liver

In the liver, the DEGs were annotated for 15 biological processes, with the most significant enrichments observed in the “single-organism process”, “cellular process”, “metabolic process”, “biological regulation”, and “response to stimulus”. Moreover, seven DEGs were associated with the “immune system process”. In total, 11 GO categories were found in cellular components, with “cell part”, “cell”, “membrane”, and “organelle” being the top four GO enrichment terms. Among molecular functions, six GO categories were identified, with “binding” and “catalytic activity” being the most enriched (Figure 3a).

#### 3.3.2. Spleen

In the spleen, 18 biological processes were noted, with the top five enrichments being similar to those in the liver. However, the spleen demonstrated more DEGs (n = 20) annotated for the “immune system process”. In total, 15 GO categories were identified in cellular components, with >100 DEGs annotated. Among molecular functions, 11 categories were identified, including “binding”, “catalytic activity”, “molecular transducer activity”, and “molecular function regulator” (Figure 3a).

#### 3.3.3. Head Kidney

In the head kidney, DEGs were annotated for 20 biological processes, with the top five GO enrichments being similar to those in the liver. Moreover, 16 DEGs were annotated for the “immune system process”. In total, 15 GO categories were found in cellular components, with “cell part”, “cell”, “membrane”, and “organelle” being the top four GO enrichment. Among molecular functions, 15 GO categories were identified, with the main enrichment noted in “binding”, “catalytic activity”, “molecular transducer activity”, and “transporter activity” (Figure 3a).

#### 3.3.4. Trunk Kidney

In the trunk kidney, 19 biological processes were observed, with 48 DEGs being associated with “immune system process”. Moreover, 16 GO categories were found in cellular components, with “cell part”, “cell”, “membrane”, and “organelle” being the top four GO enrichment, and more than 300 DEGs were annotated. Among molecular functions, 12 GO categories were identified, with the main enrichment noted in “binding”, “catalytic activity”, “molecular transducer activity”, and “transporter activity” (Figure 3a).

#### 3.3.5. GO Terms in Four Tissues

A detailed comparison of immune-related terms across the four tissues revealed a total of 110 distinct terms (Table S5). Figure 3b depicts a Venn diagram of the distribution and overlap of these terms among tissues, with the liver, spleen, head kidney, and trunk kidney showing 18, 28, 9, and 40 unique terms, respectively. These terms encompassed a range of functions, including virus response, innate immunity, cytokine regulation, cell death, and adaptive immunity. Notably, the “cytokine-mediated signaling pathway” was coenriched in all four tissues (Figure 3b). The enrichment of the “regulation of p38MAPK cascade” was observed in the liver, spleen, and trunk kidney (Figure 3b). The liver, head kidney, and trunk kidney shared terms related to cytokines, namely the “heme biosynthetic process” and “immune response”, whereas the liver and spleen demonstrated common endocytosis-related terms (Figure 3b). The liver and trunk kidney exhibited enrichment of a total of three immune-related terms, of which two were associated with cytokine-related processes and one was related to virus-related mechanisms (Figure 3b). The spleen, head kidney, and trunk kidney shared 15 immune-related terms, including those related to apoptosis, inflammation, and adaptive immunity (Figure 3b). Furthermore, the spleen and trunk kidney displayed coenrichment of 13 immune-related terms including those related to apoptosis, endocytosis, and interleukin activity. The head and trunk kidneys had nine significantly enriched immune-related terms, including those associated with apoptosis, autophagy, cytokine, immune cell, and immunoregulation (Figure 3b).

### 3.4. KEGG Enrichment of DEGs in Four Tissues

KOBAS was employed to perform a comprehensive KEGG pathway enrichment analysis of DEGs in the four tissues from GCRV-infected barbel chub. This analysis revealed a substantial enrichment of KEGG pathways, with 220 pathways being significantly enriched across the tissues (corrected *p* < 0.05). The liver, spleen, head kidney, and trunk kidney demonstrated significant enrichment of 23, 145, 121, and 172 KEGG pathways, respectively. The top 20 KEGG pathways were visualized using bubble charts for subsequent analysis. The significantly enriched KEGG pathways are listed in Table S6.

#### 3.4.1. Liver

**Figure 4:**
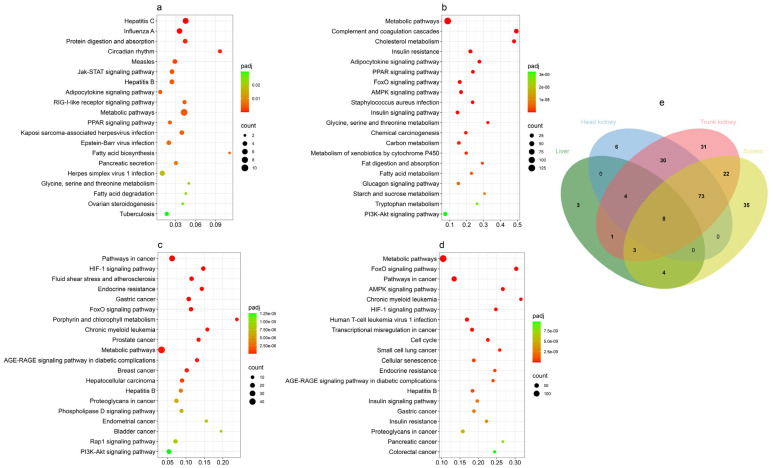
(**a**–**d**) Bubble charts of the top 20 enriched KEGG pathways in the liver (**a**), spleen (**b**), head kidney (**c**), and trunk kidney (**d**) after GCRV infection. The *x*-axis denotes gene ratios, the left *y*-axis presents pathway names, and the right *y*-axis denotes adjusted *p* values and counts. (**e**) Venn diagram of top KEGG pathways in the liver (green), spleen (blue), head kidney (red), and trunk kidney (yellow) after GCRV infection. The values represent the numbers of GO terms in the corresponding tissues.

In the liver, KEGG pathway analysis delineated four distinct categories: “human diseases”, “organismal systems”, “metabolism”, and “environmental information processing”, each represented by eight, seven, four, and one pathway, respectively. Notably, “hepatitis C” and “influenza A” emerged as the most significant pathways, whereas “metabolic pathways” featured the highest number of DEGs. Moreover, pivotal immune-related pathways, specifically the “RIG-I-like receptor signaling pathway” and “Jak-STAT signaling pathway”, were identified (Figure 4a).

#### 3.4.2. Spleen

In the spleen, the categorization of KEGG pathways paralleled that of the liver. “Organismal systems” and “metabolism” each presented seven pathways, whereas “environmental information processing” and “human diseases” each comprised three pathways. “Metabolic pathways” appeared as the most significant and gene enriched. “Complement and coagulation cascades” were significant among immune-related pathways. Furthermore, numerous metabolism-related pathways were observed; in particular, they encompassed lipid metabolism-related pathways (“fat digestion and absorption”, “adipocytokine signaling pathway”, “fatty acid metabolism”, and “cholesterol metabolism”), insulin-related pathways (“insulin resistance”, “insulin signaling pathway”, and “glucagon signaling pathway”), amino acid metabolism-related pathways (“tryptophan metabolism” and “glycine, serine and threonine metabolism”), and sugar metabolism-related pathways (Figure 4b).

#### 3.4.3. Head Kidney

The head kidney analysis revealed three main categories of KEGG pathways: “human diseases”, “environmental information processing”, and “metabolism”, with 13, 5, and 2 pathways, respectively, annotated in each. “Pathways in cancer” was the most significant pathway category. The “human diseases” category predominantly featured pathways related to cancer, cardiovascular diseases, virus diseases, and metabolic signaling, with metabolic signaling pathways including “metabolic pathways” (which displayed the largest number of DEGs in the trunk kidney) and “porphyrin and chlorophyll metabolism”. Notably, numerous pathways, such as those related to lipid metabolism, glucose metabolism, inflammation, and apoptosis, could not be visually represented in the bubble plots (Figure 4c).

#### 3.4.4. Trunk Kidney

In the trunk kidney, the top 20 pathways were categorized into five groups. The “human diseases” category had 15 pathways. Within this category, nine pathways pertained to cancer, three to viral infections, and three to endocrine and metabolic-related disorders. “Organismal systems” included three pathways, two of which were related to the endocrine system. The “cellular processes” category featured two pathways associated with cell growth and death (Figure 4d). Notably, numerous critical pathways, including those involved in lipid metabolism, glucose metabolism, amino acid metabolism, inflammation, and cell death, were identified but not represented in the bubble map.

#### 3.4.5. KEGG Intersection Analysis in All Tissues

The KEGG enrichment analysis results of all tissues were visualized using Venn diagrams (Table S7). The liver displayed three unique pathways: two “human diseases” pathways linked to bacterial and viral infections, and the “RIG-I-like receptor signaling pathway” in PRRs (Figure 4e). The spleen had 35 unique pathways, including six immune-related pathways, as well as numerous metabolic-related pathways (Figure 4e). In the head kidney, six unique pathways were identified, five of which were immune-related and one associated with “protein processing in the endoplasmic reticulum” (Figure 4e). The trunk kidney presented 31 unique pathways, predominantly related to metabolism, cell death, cell adhesion, development, and nucleic acid repair and degradation (Figure 4e). Four pathways were shared between the liver and spleen, including two pathways related to “human diseases” and two related to metabolism (Figure 4e). “Circadian rhythm” was identified in both the liver and trunk kidney (Figure 4e). In total, 22 shared pathways in the spleen and trunk kidney included metabolic pathways, “apoptosis” pathways, endocrine-related pathways, and pathogen infection-related “human diseases” pathways (Figure 4e). In total, 30 shared pathways were found in the trunk and head kidneys, spanning categories such as human diseases, metabolism, cell adhesion, and various immune-related pathways (Figure 4e). The spleen, head kidney, and trunk kidney were noted to share many pathways, particularly in those associated with metabolism, cancer, cell death, and inflammation (Figure 4e). Across all tissues, eight identical pathways were identified, of which three were related to human diseases virus infections and five to lipid metabolism (Figure 4e).

### 3.5. Representative Immune DEGs in Four Tissues

Via comprehensive GO and KEGG enrichment analyses, 1, 6, 13, and 14 distinct pathways related to viral immunity were identified in the liver, spleen, head kidney, and trunk kidney, respectively. These pathways encompassed various aspects of immune response, including inflammation-related pathways (e.g., complement and coagulation cascades, PRR pathways, and cytokine-related pathways), as well as pathways involved in apoptosis, autophagy, and general immune responses. The genes within these identified pathways were subjected to further detailed analysis.

#### 3.5.1. Complement and Coagulation Cascade Response Patterns

**Figure 5:**
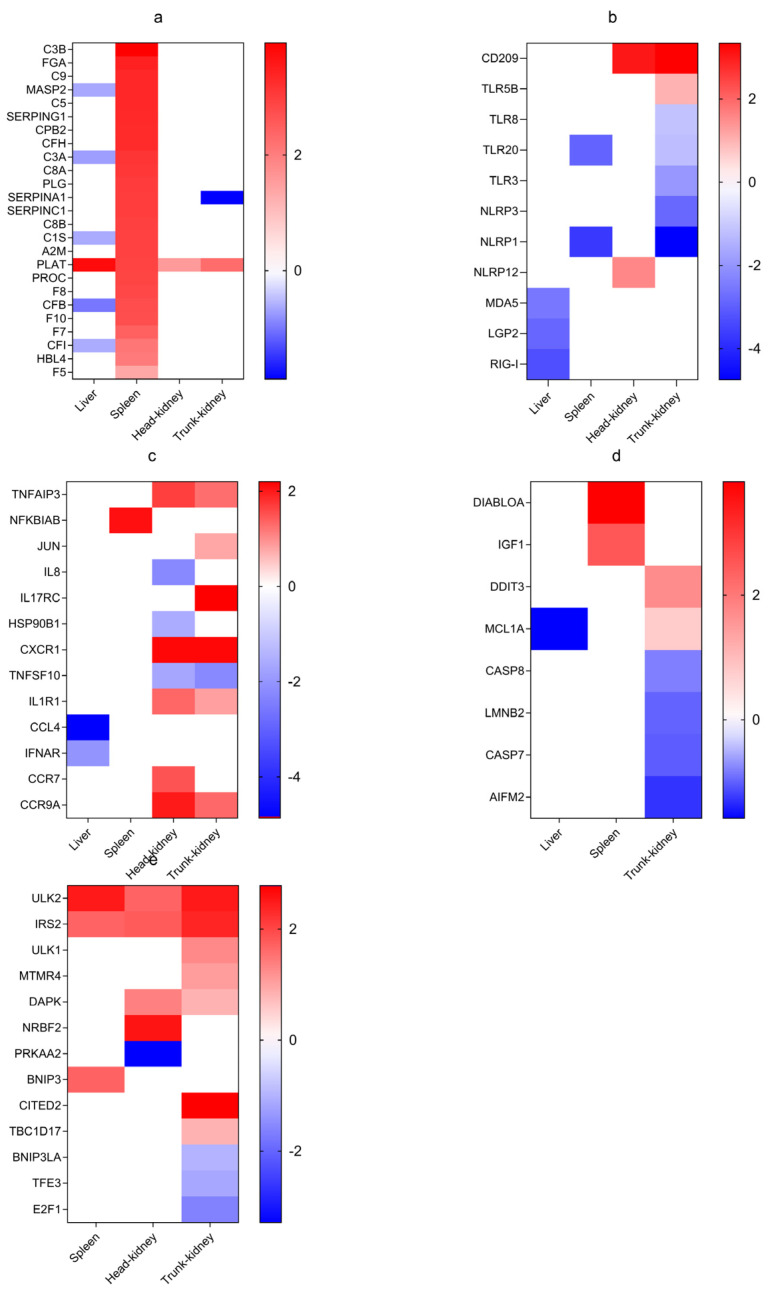
Heatmaps of DEGs enriched in the liver, spleen, head kidney, and trunk kidney based on their log_2_(FC) values. (**a**) Complement and coagulation cascade genes; (**b**) PRRs; (**c**) Cytokine-related genes; (**d**) Apoptosis-related genes; (**e**) Autophagy-related genes.

In the spleen, genes related to “complement and coagulation cascades” were expressed predominantly, with many being notably activated and upregulated. These include complement component 1S (*C1S*), mannose-binding lectin (*MBL*), mannose-binding lectin-associated serine protease 2 (*MASP2*), complement component 3A (*C3A*), complement component 3B (*C3B*), complement component 5 (*C5*), and complement component 9 (*C9*) in the complement cascade and fibrinogen alpha chain (*FGA*), serpin family I member 1 (*SERPINI1*), serpin family F member 2B (*SERPINF2B*), and plasminogen (*PLG*) in the coagulation cascade (Figure 5a). Moreover, a subset of DEGs, particularly complement cascade genes (mannose-binding lectin-associated serine protease 1/2 (*MASP1/2*), complement component 3 (*C3*), *C3A*, *C1S*, complement factor B (*CFB*), and complement factor I (*CFI*)), were downregulated in the liver, whereas the coagulation cascade gene plasminogen activator, tissue (*PLAT*) was upregulated across all four tissues. Notably, expression of the coagulation cascade gene Serpin Family A Member 1 (*SERPINA1*) was suppressed in the trunk kidney (Figure 5a).

#### 3.5.2. PRR Pathway Response Patterns

PRR pathways were identified in all four tissues, with the highest and lowest number of DEGs noted in the trunk kidney and liver, respectively. Notably, only toll-like receptor 5B (*TLR5B*), NLR family pyrin domain containing 12 (*NLRP12*), and DC-SIGN (*CD209*) were upregulated in both the head and trunk kidneys (Figure 5b). Additionally, eight DEGs, including *MDA5*, laboratory of genetics and physiology 2 (*LGP2*), retinoid-inducible gene 1 (*RIG1*), toll-like receptor 3 (*TLR3*), toll-like receptor 20 (*TLR20*), toll-like receptor 8 (*TLR8*), NLR family pyrin domain containing 1 (*NLRP1*), and NLR family pyrin domain containing 3 (*NLRP3*), were observed to be downregulated across all tissues (Figure 5b).

#### 3.5.3. Cytokine-Related Pathway Response Patterns

In the liver, *CCL4* and *IFNAR* were downregulated, whereas *NFΚBIAB* was upregulated in the spleen (Figure 5c). Both head kidney and trunk kidney demonstrated similar expression patterns, with TNF-alpha-induced protein 3 (*TNFAIP3*), C-X-C chemokine receptor 1 (*CXCR1*), interleukin 1 receptor type 1 (*IL1R1*), and C-C motif chemokine receptor 9A (*CCR9A*) being downregulated. TNF superfamily member 10 (*TNFSF10*) was exclusively downregulated in the head kidney. In contrast, C-C motif chemokine receptor 7 (*CCR7*) was upregulated in the head kidney, whereas in the trunk kidney, interleukin 8 (*IL8*) and heat shock protein 90 beta family member 1 (*HSP90B1*) were downregulated, and Jun proto-oncogene (*JUN*) and interleukin 17 receptor C (*IL17RC*) were upregulated (Figure 5c).

#### 3.5.4. Apoptosis Response Patterns

Eight DEGs were identified in the apoptosis pathway. In the liver, myeloid cell leukemia 1A (*MCL1A*) was downregulated, whereas diablo IAP-Binding mitochondrial protein A (*DIABLOA*) and insulin-like growth factor 1 (*IGF1*) were upregulated in the spleen. The trunk kidney exhibited seven genes, with caspase-7 (*CASP7*), caspase-8 (*CASP8*), lamin B2 (*LMNB2*), and apoptosis-inducing factor mitochondrial 2 (*AIFM2*) being downregulated and DNA damage induced transcript 3 (*DDIT3*) and *MCL1A* being upregulated. Notably, none of the downregulated apoptosis-related genes had a log_2_(FC) of <−2, whereas two upregulated genes in the spleen demonstrated a log_2_(FC) of >2 (Figure 5d). GO enrichment analysis revealed numerous apoptosis-related genes in the head kidney; however, KEGG enrichment analysis did not demonstrate the presence of any enriched apoptosis pathways.

#### 3.5.5. Autophagy Response Patterns

In the autophagy-related pathways, unc-51 Like autophagy activating linase 2 (*ULK2*), insulin receptor substrate 2 (*IRS2*), and Bcl-2/adenovirus E1B 19kDa interacting protein 3 (*BNIP3*) were upregulated in the spleen. In contrast, AMP-activated protein kinase subunit alpha 2 (*PRKAA2*) was downregulated in the head kidney, and BCL2 Interacting Protein 3 Like A (*BNIP3LA*), transcription factor E3 (*TFE3*), and E2F transcription factor 1 (*E2F1*) were downregulated in the trunk kidney. Notably, three genes were linked to mitochondrial autophagy pathway regulation, whereas the remaining genes demonstrated upregulation (Figure 5e).

### 3.6. Verification of DEGs with qRT-PCR

In total, 11 DEGs were randomly selected for qRT-PCR to validate our RNA-seq data and gene expression profiles (Figure S1). The gene expression trends were consistent with the RNA-seq results, confirming the reliability of our transcriptomic data in this study.

## 4. Discussion

GCRV is associated with high infection and mortality rates in various members of the carp family. Additionally, it can also infect barbel chub; however, unlike grass carp, barbel chub exhibits a low mortality rate after GCRV infection. Understanding the immune mechanisms in barbel chub is, therefore, crucial for the advancement of breeding strategies aimed at enhancing GCRV resistance in grass carp.

The liver, spleen, head kidney, and trunk kidney are integral to the immune response in fish. The liver, particularly rich in natural killer cells, plays a crucial role in immune function [21,22]. Similarly, the spleen, a secondary lymphoid organ, is pivotal in adaptive immunity initiation [23]. In bony fish, the kidney is divided into head and trunk kidneys. Recent studies have suggested that the head kidney, rich in lymphocytes, functions similarly to mammalian bone marrow, functioning both as a primary and secondary lymphoid organ [23,24]. Furthermore, the trunk kidney, primarily an excretory organ, displays significant immune capabilities. For instance, in grass carp, the trunk kidney demonstrated strong antipathogen responses to GCRV and bacterial PAMP infections [25]. Similarly, in rainbow trout, the trunk kidney exhibits enhanced immune responses during infectious hemolytic necrosis virus infection compared with the head kidney [26]. In the current study, the number of DEGs identified in the liver, spleen, head kidney, and trunk kidney of barbel chub at 120 h after GCRV infection were 139, 970, 867, and 2374, respectively. This finding is in disagreement with the DEG distribution results observed in grass carp tissues after a similar duration of GCRV infection. The discrepancy may be attributable to variations in sequencing depth and transcriptome assembly quality [18].

Studies have indicated that the host immune system adjusts its response mechanisms according to different stages of viral infection. For instance, Wang et al. [18] found that 5 days after GCRV infection, grass carp demonstrated a significant increase in DEGs associated with PRRs, cytokines, phagosomes, lysosomes, and other pathways. Moreover, secondary infection of GCRV-resistant grass carp demonstrated that 5 days after GCRV infection, pathways related to antigen processing and presentation, toll-like receptor (TLR) signaling, and natural killer cell-mediated cytotoxicity were significantly activated [27]. These findings suggest that the grass carp immune system exhibits a robust immune response, particularly around GCRV infection day 5. Assessment of the immune response of barbel chub immune tissues within 5 days after GCRV infection can aid in elucidating the anti-GCRV immune mechanisms of barbel chub, providing a theoretical basis for further research on the immune molecular mechanisms underlying barbel chub resistance to GCRV.

PRRs act as sentinels in immune responses, rapidly initiating immune response on PAMP detection and facilitating cytokine secretion in immune cells [28]. They have a critical role in orchestrating immune responses in fish. TLRs, such as TLR22, are upregulated in grass carp during GCRV infection [29]. In black carp, LGP2 is pivotal in responding to spring viremia of carp virus and GCRV [30], whereas in grouper, MDA5 regulates proinflammatory cytokine secretion and impedes viral infections such as Singapore grouper iridovirus and red-spotted grouper nervous necrosis virus [31]. Notably, PRR downregulation during pathogen infection is a crucial step that aids the completion of the associated biological processes. For instance, vesicular stomatitis virus, an oncolytic virus, can infect cancer-associated fibroblasts, downregulating RIG-I expression and increasing susceptibility to viral infection, thus aiding in tumor cell elimination [32]. Similarly, the small intestinal barrier, rich in beneficial bacterial components and sensitive to injury, modulates TLR expression to avert tissue damage from commensal microorganisms [33]. These findings indicate that reduced PRR expression can enable pathogen cell invasion and prevent unwarranted tissue damage. In the present study, we identified 10 differentially expressed PRRs in barbel chub after GCRV infection, with only TLR5b (TLR pathway) and CD209 (lectin receptor pathway) being upregulated. The remaining eight PRRs were downregulated, a pattern contrasting with that observed in GCRV-infected grass carp. This suggests that in barbel chub, PRRs may not directly recognize the virus, potentially activating antiviral immune responses via alternative pathways.

Cytokines, a diverse group of low-molecular-weight proteins, are fundamental in intercellular communication and immune regulation [3]. In response to pathogen invasion, immune cells release cytokines, triggering inflammatory responses in tissues. For instance, Interleukin-1β can modulate the interferon response in grass carp, impede GCRV replication, and elicit a robust inflammatory reaction [34]. Interleukin-8 is essential for initiating inflammatory responses and plays a significant role in immune defense against viral hemorrhagic septicemia virus [35]. In grass carp with GCRV infection, cytokine interactions and the Interleukin-17 pathway, among other cytokine-related pathways, demonstrate notable enrichment [36]. However, recent research indicates that cytokine-induced aberrant inflammatory responses during disease progression can be harmful to the host [37]. For instance, in Chinese soft-shelled turtles infected with *Aeromonas hydrophila*, dysregulation of cytokine expression leads to hemorrhagic symptoms [38]. Furthermore, GCRV infection triggers Interleukin-6 and tumor necrosis factor-α expression via C5a/C5aR signaling in grass carp, initiating inflammatory responses that result in muscle hemorrhage and liver injury [39]. These findings highlight the critical role of cytokines in orchestrating immune responses to pathogens; however, excessive cytokine release can cause significant tissue damage. In the current study, we identified specific cytokine-related genes among the DEGs in barbel chub after GCRV infection. Notably, after 120 h of infection, inflammation-related genes were upregulated. A subset of proinflammatory cytokines was downregulated, whereas genes related to inflammation suppression were upregulated. Therefore, the balanced inflammatory and immune responses observed in barbel chub 120 h after GCRV infection may explain its atypical symptom presentation. This balance appears to be a key factor in the fish’s immune strategy against GCRV, underscoring the complex interplay between cytokine regulation and immune response.

Complement and coagulation cascades are crucial in regulating inflammation, activating adaptive immunity, and eradicating pathogens [40]. Viral infiltration activates these systems in the host. The complement system, specifically, generates anaphylatoxins C3a, C4a, and C5a, which bind selectively to receptors on various cell membranes, prompting immune cells to release cytokines and initiate tissue inflammation. Neutrophil recruitment to inflammation sites outside blood vessels is mediated by β2 integrin [41,42,43,44]. Moreover, the terminal complement system pathway directly interacts with viruses via the membrane attack complex, leading to viral neutralization [45]. Recent studies have shown that antibodies and complement C4b binding to nonenveloped viruses can directly hinder viral infection [46]. Furthermore, coagulation cascades significantly contribute to immune responses. The current results suggest that the coagulation and immune systems are derived from a common precursor and thereby provide a theoretical foundation for the involvement of the coagulation system in immune responses [47]. Coagulation factors within the cascade can activate immune cells and promote proinflammatory cytokines, thus directly affecting immune cell function. Moreover, fibrin can act as a scaffold for immune cells, enhancing immune responses [48]. Li et al. [49] found that overexpression of the coagulation factor SERPINA1 inhibits GCRV infection. After GCRV infection, grass carp spleens demonstrated impaired complement and coagulation cascades and reduced transcription of key genes such as the C3, C5, and complement factor 7 genes, indicating GCRV’s ability to hinder these cascades in grass carp [16]. In this study, KEGG analysis of DEGs indicated a significant focus on inflammation-related pathways, including complement and coagulation cascades, PRRs, T-cell receptor pathways, and Th1 and Th2 cell differentiation. Closer examination of these pathways revealed that complement and coagulation cascades were the dominant annotated pathways in the spleen, with upregulated DEGs such as SERPINA1, C1S, C3A, C3B, C5, and C9. Notably, in barbel chub, normal activation of these cascades is correlated with increased resistance to GCRV infection. This evidence underscores the essential role of complement and coagulation cascades in the immune response process in barbel chub.

Apoptosis, a form of programmed cell death, is essential in maintaining cellular homeostasis during normal development and aging; it also acts as an innate defense against infectious diseases [50]. In the presence of pathogens, apoptotic cells are phagocytosed by macrophages and other phagocytic cells rather than collapsing [7]. Apoptosis can be induced by many molecular pathways, such as PRRs and the complement system. Viral infections, for instance, activate PRRs, leading to the induction of transcription factors nuclear factor kappa-B (NF-κB) and Interferon regulatory Factor 3/7 and subsequently triggering apoptosis via NF-κB signaling [51]. Complement component C1q, when deposited on apoptotic cells, induces phagocytosis by monocytes [52]. In addition, anaphylatoxins from the complement system can stimulate tumor necrosis factor and IL production, promoting apoptotic processes [39]. These findings demonstrate that viral infections often induce apoptosis and inflammatory responses. However, some pathogens manipulate apoptosis to facilitate infection. Viruses can use apoptosis for various purposes, including enhancing virus shedding and transmission, hindering antigen presentation by host cells, and eliminating uninfected bystander and immune cells [7]. For instance, the replication efficiency of influenza viruses depends on apoptosis regulated by Bcl-2 agonist of cell death [53]. In the current study, we observed the downregulation of several apoptosis-related genes in the liver and trunk kidney of GCRV-infected barbel chub, including caspase-7 and caspase-8. In contrast, we detected upregulation of apoptosis-related genes in the spleen, possibly linked to normal complement system activation. These findings indicated that GCRV infection leads to suppression of the apoptosis mechanism in barbel chub. In contrast, we detected upregulation of apoptosis-related genes in the spleen, possibly linked to normal complement system activation. These findings indicated that although barbel chub initiates apoptotic activation on GCRV infection, an overall inhibition of apoptosis occurs. We, therefore, hypothesize that in barbel chub, GCRV stimulation leads to a tightly regulated, selective apoptotic process, suggesting the presence of a sophisticated mechanism of immune response modulation.

Autophagy, a critical cellular process, is instrumental in preserving internal homeostasis, particularly by managing the intricate balance between inflammation and immunity [54]. In mammals, the mechanistic aspects of autophagy in immunity involve canonical and noncanonical pathways, with Autophagy-Related 14 (*ATG14L*), Autophagy-Related 13 (*ATG13*), and nc-51 Like Autophagy-Activating Kinase 1 (*ULK1*) driving the canonical pathway, selectively targeting various cargoes [6]. Recent studies have highlighted that autophagy primarily plays an anti-inflammatory role during inflammatory responses [6]. However, research on the role of autophagy in fish immune responses. Rao et al. [55] demonstrated that ROS-induced autophagy can hinder GCRV infection at the cellular level. In addition, autophagy has been found to be crucial in protecting CIK cells in grass carp from excessive inflammatory responses after GCRV infection [56]. In *Epinephelus* spp., the autophagy genes Autophagy-Related 5 (*ATG5)*, Autophagy-Related 12 (*ATG12)*, and Autophagy-Related 16 Like 1 (*ATG16L1*) were observed to reduce inflammatory levels [56]. These findings suggest that autophagy can aid the host’s antiviral immune response. In the current study, we noted autophagy pathway activation in the spleen, head kidney, and trunk kidney of barbel chub with GCRV infection. *ULK2* is a canonical autophagy-related DEG found in these tissues, indicating that canonical autophagy contributes to the immune response against GCRV in barbel chub. We also observed that inflammation levels remained balanced throughout the infection. Based on these observations and the survival data, autophagy may play a proactive role in the immune response during GCRV infection in barbel chub and safeguard tissues from excessive inflammation. In other words, in barbel chub, autophagy may actively assist immune responses and protect tissues from excessive inflammation during GCRV infection.

## 5. Conclusions

**Figure 6:**
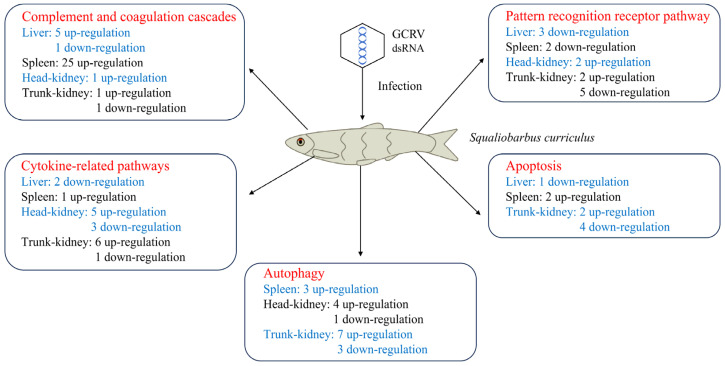
Distinctive mechanisms underlying immune responses to GCRV infection in barbel chub.

In this study, we used RNA-seq to construct a transcriptome atlas for four principal immune tissues of GCRV-infected barbel chub: the liver, spleen, and head and trunk kidneys. Our analysis focused on the molecular immune response of barbel chub to the GCRV challenge. The gene expression data suggested that during the immune response, activation of complement and coagulation cascades, suppression of PRRs and apoptosis, and the antiviral role of autophagy collectively contribute to GCRV resistance in barbel chub (Figure 6). Although these observations have not yet been functionally validated, the comparative transcriptomic data obtained here provide valuable insights. They set the foundation for further research on molecular immune mechanisms against GCRV infection in barbel chub and may inform breeding strategies aimed at enhancing GCRV resistance in grass carp via grass carp–barbel chub hybridization.

## Data Availability

The RNA-seq raw data has been submitted to the GenBank database with the accession number PRJNA1061050.

## Supplementary Materials

The following supporting information can be downloaded at: https://www.mdpi.com/article/10.3390/biology13040214/s1.
