# Supplementary material for: Comparative Transcriptomics Analysis Reveals Unique Immune Response to Grass Carp Reovirus Infection in Barbel Chub (Squaliobarbus curriculus)
Source: Biology (Basel). 2024 Mar 25;13(4):214. doi: 10.3390/biology13040214 (PMC11047996; doi:10.3390/biology13040214)
Supplement: Supplementary file 1 [file biology-13-00214-s001.zip › Table S1.docx]

Table S1: Primers used for qPCR experiments.

| Primer Name | 5’-3’ |
| --- | --- |
| β-actin-qF | CCTTCTTGGGTATGGAATCTTG |
| β-actin-qR | AGAGTATTTACGCTCAGGTGGG |
| C1S-qF | ACTGTGGAGATCCAAAGCCG |
| C1S -qR | CTTCTGCCCATTTCCGGTCT |
| MASP2-qF | AGGGAGTACGGCCCATTTTG |
| MASP2-qR | GAGGAGTCACAGGGTTTGGG |
| MDA5-qF | AGCCTCGCAGCTCAAAATCT |
| MDA5-qR | TCGTGTGGTGGCATTCATCA |
| RIG-I-qF | AGATCCTGGTGAACGCACTG |
| RIG-I-qR | CTGTGGCAGTGAGTGTTTGC |
| IRS-2-qF | GTCAGAGTTCAGACCACGCA |
| IRS-2-qR | TTGGTGACAGGAGATGTGGC |
| NFKBIAB-qF | GCGGTGGACCTTCAGAACTT |
| NFKBIAB-qR | AAGTTCCCTTAGGTGTGGCG |
| ULK-2-qF | GTGCCAAACATCCCGAGAGA |
| ULK-2-qR | GAGACCAAGCCAGAGCATGT |
| IL8-qF | GTAGCAGCCCTCATGTCTCTG |
| IL8-qR | CCTCGTTTTTGCATTGTGGC |
| Caspase7-qF | CTGACGGAGCCATACCCATC |
| Caspase7-qR | ATCGTGTCATTGGGTGGTCC |
| NLRP1-qF | CCTGTGTTTAGGTGGACCCC |
| NLRP1-qR | CCTGCCTCACAAGAACACCT |
| NLRP3-qF | AACATCAGCGCAGACAGTCA |
| NLRP3-qR | TGATTGGCTTTTCCGCTTGC |
