# Supplementary material for: Comparative Transcriptomics Analysis Reveals Unique Immune Response to Grass Carp Reovirus Infection in Barbel Chub (Squaliobarbus curriculus)
Source: Biology (Basel). 2024 Mar 25;13(4):214. doi: 10.3390/biology13040214 (PMC11047996; doi:10.3390/biology13040214)
Supplement: Supplementary file 1 [file biology-13-00214-s001.zip › Table S2.docx]

Table S2: Barbel chub transcriptomes statistical table.

| Tissue | Clean read | Clean bases (bp) | GC Content | Q30 (%) | Mapped rates |
| --- | --- | --- | --- | --- | --- |
| T25 | 23,429,188 | 6,962,784,010 | 49.70% | 86.40% | 17,129,041(73.11%) |
| T26 | 25,351,857 | 7,534,197,592 | 48.54% | 87.54% | 19,798,988(78.10%) |
| T27 | 25,440,903 | 7,557,538,606 | 48.59% | 87.80% | 19,683,067(77.37%) |
| T28 | 22,213,212 | 6,587,178,068 | 49.06% | 86.33% | 15,897,663(71.57%) |
| T29 | 29,395,049 | 8,718,255,416 | 48.03% | 86.72% | 20,868,514(70.99%) |
| T30 | 22,493,297 | 6,667,783,074 | 47.83% | 86.42% | 16,052,739(71.37%) |
| T31 | 26,639,014 | 7,908,594,630 | 47.71% | 86.27% | 18,845,665(70.74%) |
| T32 | 25,637,837 | 7,609,769,358 | 47.48% | 86.58% | 19,101,115(74.50%) |
| T33 | 22,650,068 | 6,710,539,858 | 48.86% | 86.11% | 16,219,844(71.61%) |
| T34 | 22,410,878 | 6,647,234,242 | 48.08% | 86.53% | 16,815,023(75.03%) |
| T35 | 26,530,078 | 7,875,328,686 | 47.75% | 86.56% | 19,192,613(72.34%) |
| T36 | 27,839,435 | 8,282,730,286 | 49.64% | 85.77% | 20,540,764(73.78%) |
| T37 | 24,809,057 | 7,375,910,532 | 48.09% | 85.84% | 18,271,804(73.65%) |
| T38 | 24,348,405 | 7,200,224,962 | 48.43% | 87.29% | 18,449,800(75.77%) |
| T39 | 27,842,786 | 8,258,796,120 | 48.75% | 86.78% | 20,813,654(74.75%) |
| T40 | 27,550,749 | 8,175,384,206 | 48.84% | 86.72% | 19,332,306(70.17%) |
| T41 | 25,799,281 | 7,667,028,466 | 48.74% | 86.76% | 19,690,126(76.32%) |
| T42 | 25,274,779 | 7,520,879,600 | 47.37% | 85.87% | 17,575,215(69.54%) |
| T43 | 24,534,728 | 7,288,851,218 | 47.69% | 85.01% | 17,129,486(69.82%) |
| T44 | 24,539,275 | 7,296,795,236 | 47.28% | 85.09% | 17,265,916(70.36%) |
| T45 | 21,977,711 | 6,536,129,790 | 48.14% | 85.29% | 16,805,109(76.46%) |
| T46 | 21,759,256 | 6,458,697,926 | 49.23% | 85.25% | 15,150,520(69.63%) |
| T47 | 20,669,088 | 6,140,964,300 | 47.48% | 85.17% | 14,784,180(71.53%) |
| T48 | 21,540,014 | 6,395,389,052 | 48.03% | 85.40% | 15,343,015(71.23%) |

Note: Control group: T25, T26, T27: Liver; T28, T29, T30: Spleen; T31, T32, T33: Head kidney; T34, T35, T36: Trunk kidney. Experiment group: T37, T38, T39: Liver; T40, T41, T42: Spleen; T43, T44, T45: Head kidney; T46, T47, T48: Trunk kidney. Clean read: total number of pair-end reads in clean date. Mapped rates: The proportion of Mapped Reads in Clean Reads.
