# Supplementary material for: Comparative Transcriptomics Analysis Reveals Unique Immune Response to Grass Carp Reovirus Infection in Barbel Chub (Squaliobarbus curriculus)
Source: Biology (Basel). 2024 Mar 25;13(4):214. doi: 10.3390/biology13040214 (PMC11047996; doi:10.3390/biology13040214)
Supplement: Supplementary file 1 [file biology-13-00214-s001.zip › Table S3.docx]

Table S3: DEGs statistics in GCRV-infected tissues of barbel chub

| Tissue | All_DEGs | Upregulation gene | Downregulation gene |
| --- | --- | --- | --- |
| Liver | 139 | 20 | 119 |
| Spleen | 970 | 869 | 101 |
| Head kidney | 867 | 653 | 214 |
| Trunk kidney | 2374 | 1565 | 809 |
